# Sustainability in Cataract and Refractive Surgery: Current Challenges and Future Perspectives

**DOI:** 10.1155/joph/4632626

**Published:** 2025-10-16

**Authors:** Pier Luigi Surico, Uday Pratap Singh Parmar, Chi-Chin Sun, Paolo Lanzetta

**Affiliations:** ^1^Department of Sense Organs, Sapienza University, Rome, Italy; ^2^Department of Ophthalmology, Chang Gung Memorial Hospital, Keelung, Taiwan; ^3^Department of Ophthalmology, Government Medical College and Hospital, Chandigarh, India; ^4^Department of Medicine-Ophthalmology, University of Udine, Udine, Italy; ^5^Istituto Europeo di Microchirurgia Oculare-IEMO, Udine, Italy

**Keywords:** carbon footprint, cataract, ophthalmic surgery, refractive surgery, sustainability

## Abstract

Cataract and refractive surgery are integral to improving vision health and quality of life; however, their growing environmental impact poses significant concerns. These procedures contribute notably to medical waste, excessive energy consumption, and pharmaceutical overuse, amplifying the carbon footprint of health care. Key environmental challenges include the dependence on single-use surgical instruments, high energy demands from laser and phacoemulsification systems, and the waste associated with medication disposal and packaging. Although some sustainable initiatives, such as reusable surgical tools, biodegradable packaging, and optimized pharmaceutical usage, have been introduced, widespread implementation remains limited. This review investigates the environmental impact of ophthalmic surgery, assesses current sustainable practices, and highlights emerging eco-friendly innovations. Notable advancements include energy-efficient surgical devices, electronic instructions for use, optimized postoperative protocols, and regulatory policies aimed at promoting green hospital systems. However, further research into life cycle assessments, carbon footprint evaluations, and regulatory reforms will be crucial to advancing global sustainable practices without compromising patient care and surgical outcomes.

## 1. Introduction

Despite its crucial role in society, the healthcare sector significantly contributes to environmental pollution, which, in turn, affects public health. Research by Klangsin and Harding highlights that medical waste has become a leading global pollutant, playing a critical role in disease transmission and negatively impacting air, water, and soil quality within and around healthcare facilities [1]. The environmental footprint of health care was further amplified during the COVID-19 pandemic, which also had a significant impact on eye care [2]. The surge in healthcare activities, coupled with the widespread use of personal protective equipment (PPE), diagnostic tools, and vaccines for SARS-CoV-2, led to a dramatic rise in medical waste generation [3].

Sustainability in health care refers to a comprehensive system that integrates various strategies to maintain, restore, and enhance human health while being environmentally, economically, and socially sustainable. A truly sustainable healthcare system operates in harmony with both the human body and the broader ecosystem, ensuring long-term viability without imposing undue burdens on any key component of the system [4].

The epidemic of refractive errors, particularly myopia, is dramatically increasing in children and adolescents, leading to debilitating effects on vision and imposing a substantial social and economic cost [5]. However, aging affects the eye, and increasing life expectancy has led to a higher prevalence of cataracts, placing a significant burden on healthcare systems [6, 7].

Therefore, cataract and refractive surgery play a crucial role in improving vision health, addressing common conditions that impact millions worldwide. Refractive surgery, including LASIK, PRK, and SMILE, is widely used to correct myopia, hyperopia, and astigmatism, significantly improving quality of life and productivity [8]. New technologies, such as SILK, are rapidly expanding, particularly in Asian countries, introducing novel sustainability challenges related to energy consumption, material waste, and long-term environmental impact, while simultaneously aiming to enhance patient safety and surgical effectiveness [9]. Over the past decade, SMILE has been extensively evaluated and compared to LASIK, a well-established and successful surgical technique [8]. Additionally, phakic intraocular lenses (IOLs) provide an alternative for patients who may not be suitable candidates for corneal refractive procedures [10]. However, recent studies suggest that preventing myopia progression could potentially be more cost-effective than treating the long-term complications associated with pathologic myopia [5, 11].

Cataract surgery remains one of the most frequently performed surgical procedures globally, as cataracts continue to be the leading cause of blindness. The most common type, age-related (senile) cataract, becomes increasingly prevalent with age, affecting 3.9% of individuals aged 55–64 and rising to 92.6% in those aged 80 and above [12, 13]. Other forms include congenital and secondary cataracts. Among children, cataract prevalence is approximately 4.2 per 100,000, with the highest rates observed in Asia at 7.4 per 100,000 [14].

In the United States, more than four million cataract surgeries are performed annually, with a projected 3%–4% annual increase. By 2030, this number is expected to reach six million, highlighting the growing demand for this vision-restoring procedure [15]. Moreover, a recent study has shown that treating cataracts not only improves vision quality but also helps prevent severe, sometimes life-threatening events in older patients, such as fractures [16]. In this way, it can potentially contribute to indirectly reducing other major healthcare costs and socioeconomic burdens. A holistic approach to cataract surgery, combined with advanced technologies, aims not only to treat the disease but also to achieve precise refractive outcomes, enhancing both visual function and overall quality of life for patients [17–19].

The healthcare sector significantly contributes to climate change, with operating rooms (ORs) generating approximately 70% of the 4 billion tons of waste produced annually in the United States [20]. Globally, health care accounts for nearly 5% of greenhouse gas (GHG) emissions, a key driver of climate change [21]. In the United Kingdom, the National Health Service (NHS) alone produces an estimated 20 million tons of GHGs per year, making up 25% of all public sector emissions [22].

With nearly 30 million cataract surgeries performed worldwide each year, these ophthalmic procedures generate a substantial amount of waste. In the United States alone, these procedures produce an estimated 12,800 metric tons of waste. Each case contributes between 2.3 and 3.9 kg of discarded materials, much of which could be minimized with sustainable practices [23].

Addressing sustainability in ophthalmic surgeries is critical. Reducing waste, optimizing energy use, and implementing responsible pharmaceutical disposal can significantly decrease the environmental footprint of these life-changing procedures [24]. As the global demand for eye surgeries continues to rise, adopting eco-friendly practices will be essential for balancing patient care with environmental responsibility.

This review aims to provide a comprehensive overview of the environmental impact of cataract and refractive surgery, highlighting key challenges, existing sustainable practices, and future directions. We herein examine critical issues such as single-use surgical materials, high energy consumption, excessive pharmaceutical waste, and the carbon footprint of ophthalmic procedures, as summarized in Table 1. By analyzing the current barriers to sustainability, we underscore the need for systemic change in surgical protocols, material usage, and energy efficiency, emphasizing the role of technological innovation, interdisciplinary collaboration, regulatory policies, and industry-led initiatives.

## 2. Sustainability Issues

### 2.1. Single-Use Materials

Ophthalmic surgeries, including refractive and cataract procedures, heavily rely on disposable instruments, surgical drapes, and packaging. Strict mandates enforcing single-use materials generate significant waste and increase costs, despite a lack of proven benefit in preventing postoperative endophthalmitis (POE) [43].

Cataract surgeries, particularly phacoemulsification, generate a considerable amount of waste. One study reported that each lens extraction using phacoemulsification produced an excess of 280 g of plastic waste, 8 g of paper waste, and 78.7 g of CO_2_ emissions compared to manual small-incision cataract surgery (MSICS) [44]. Another analysis found that across 203 phacoemulsification cases, 167.965 kg of waste was produced, with 56.6% classified as clinical waste, 37.6% as general waste, and 5.8% as sharps. Encouragingly, 50.9% of the general waste was recyclable. However, waste production varied by surgeon experience, with each phacoemulsification surgery generating 0.814 kg of waste for experienced ophthalmologists and 1.086 kg for trainees. The estimated carbon footprint per case was 0.282 kg of CO_2_ equivalents [25].

A study in Austria examined variations in cataract surgery packaging, identifying 55 different package compositions with an average weight of 0.7 kg. Some packages were up to 57% lighter than others, and surgical drape sizes varied by as much as 71%. As drapes and covers account for approximately 53% of total package weight, standardizing and reducing material use could significantly cut waste. They concluded that if their eye departments adopted the lowest material-use standards, cataract surgery-related CO_2_ emissions could be reduced by 34% [26].

Packaging waste also plays a significant role in ophthalmic surgery's environmental impact. A study from the United Kingdom's NHS found that IOL packaging alone weighed 64 g per IOL, which included an extensive 70-page instruction booklet printed in multiple languages [27]. Similarly, other ophthalmic supplies such as IOL insertion cartridges, ophthalmic viscosurgical devices (OVDs), saline dropper bottles, and phacoemulsification tips contribute to unnecessary material waste.

Recent studies have highlighted the scale of paper waste associated with IOL instruction-for-use (IFU) documents. One analysis estimated that IFUs accompanying IOLs used in Europe and the United States contribute approximately 153 tons of paper waste annually. Transitioning to electronic IFUs (e-IFUs) could reduce this by 84%, saving an estimated 128 tons of paper, preventing the emission of 120 tons of carbon dioxide equivalent, and preserving over 2000 trees each year [28]. Similarly, research by Schehlein et al. found that paper IFUs account for approximately 40 g of the 61 g total weight of an IOL package. With an estimated 30 million IOLs shipped annually, the associated production and transportation result in over 5 million kilograms of carbon dioxide equivalent emissions per year [45].

### 2.2. Energy Consumption

The energy demands of ophthalmic surgeries are substantial, particularly in procedures that use advanced laser and ultrasound (US) technologies. Refractive surgeries such as LASIK, PRK, and SMILE rely on excimer and femtosecond lasers, both of which require significant power to operate. Excimer lasers, which reshape the cornea using ultraviolet (UV) pulses, necessitate a continuous high-voltage power supply, along with energy-intensive cooling systems, air filtration units, and vacuum pumps [46, 47]. Femtosecond lasers, used in LASIK for flap creation and in SMILE for lenticule extraction, operate with ultrafast pulse technology that further increases electricity consumption [48, 49].

Similarly, cataract surgery, particularly phacoemulsification, is an energy-intensive procedure. Phacoemulsification machines use ultrasonic energy to break apart and extract the lens, requiring significant electrical input for irrigation, aspiration, and probe operation [50]. These systems must also maintain a sterile surgical environment, further adding to their energy footprint. Studies comparing different phacoemulsification techniques have found that phacofragmentation chop consumes considerably less cumulative dispersed energy (CDE) than other methods, such as divide-and-conquer or ultrachopper techniques, suggesting potential avenues for energy efficiency improvements [51]. In contrast, MSICS operates with minimal reliance on electricity, making it a more sustainable alternative, particularly in resource-limited settings [52]. However, in many regions, phacoemulsification remains the preferred approach due to its precision and faster recovery times, despite its higher energy requirements.

Beyond surgical techniques, sterilization protocols also contribute to energy consumption. Many healthcare facilities use flash autoclaving, also referred to in the United States as Immediate Use Sterile Supplies (IUSS). This method requires instruments to be sterilized and used on the same day, as they cannot be stored. Although intended to enhance patient safety, the necessity of such regulations remains scientifically unproven. Meanwhile, the process increases energy demands and operational costs, adding to the environmental burden of ophthalmic surgical procedures [41].

Additionally, we must consider that the manufacturing of IOLs and phakic lenses poses significant sustainability challenges. IOL production is an energy-intensive process that involves polymer synthesis, precision molding, and sterilization [53]. IOL optics are predominantly made from polymers, categorized into two main types: acrylic and silicone [53]. Acrylic lenses are further divided into rigid variants composed of polymethyl methacrylate (PMMA) and foldable types manufactured from either hydrophobic or hydrophilic acrylic materials, depending on their water content [54]. These primary materials—medical-grade acrylics and silicones—undergo extensive processing, contributing to the industry's environmental footprint.

### 2.3. Pharmaceutical Waste: Impact of Preoperative, Intraoperative, and Postoperative Medications

The use of preoperative, intraoperative, and postoperative medications in cataract surgery contributes significantly to both financial and environmental waste. A substantial portion of these medications, including antibiotics, steroids, and mydriatics, are discarded after single use, leading to unnecessary costs and GHG emissions.

A study by Tauber et al. analyzing four U.S. surgical facilities found that, on average, $148 worth of medication was discarded per cataract surgery [55]. Given that approximately 3.8 million cataract procedures are performed annually in the United States, this equates to an estimated $560 million in wasted medications each year. The associated carbon footprint is also considerable—each cataract case results in about 30 kg of CO_2_ emissions from discarded pharmaceuticals, amounting to 105,000 metric tons of CO_2_ emissions nationwide per year [55]. Similarly, a 2013 analysis by Morris et al. on carbon emissions from cataract surgery within the United Kingdom's NHS found that pharmaceuticals contributed to 18% of the procedure's overall emissions and were second only to medical equipment supply chains [27]. Tauber and colleagues also reported that nearly half of all medications (and two-thirds of topical drugs) were discarded after a single use, leading to an estimated $195,000 in annual waste per facility. Eye drops were the most frequently discarded, with 65.7% of their volume left unused, compared to 24.8% for injectable medications and 59.9% for systemic drugs [55]. Given that most eye drop bottles contain 5 to 15 mL, yet only a few drops are used per patient, opening a new bottle for each procedure leads to significant wastage. The cumulative impact of pharmaceutical waste across multiple facilities results in substantial CO_2_ emissions. Monthly unused medication volumes at various centers led to estimated emissions ranging from 418 kg to 2498 kg CO_2_ equivalent per center. Annually, this waste was estimated to generate between 23,000 and 105,000 metric tons of unnecessary CO_2_ emissions in the United States [55].

Despite the scale of waste, current policies at ambulatory surgery centers (ASCs) often mandate premature disposal of multidose eye drop bottles. According to a 2021 survey by the Outpatient Ophthalmic Surgery Society (OOSS), 72% of ASCs discarded partially used bottles at the end of the month, 9% at the end of the day, and only 12% used them until their labeled expiration date [56, 57]. Similarly, a 2020 survey by the Ophthalmic Instrument Cleaning and Sterilization (OICS) Task Force found that although 26% of ophthalmologists already sent topical pharmaceuticals home with patients postoperatively, 67% were open to this practice if regulations permitted it [27]. However, only 4% were opposed to the idea. Despite this willingness, restrictive facility policies (49%), state regulations (23%), and pharmacy constraints often prevented implementation [58].

A qualitative study of four U.S. surgical sites further highlighted inefficiencies in medication management. In some cases, surgeons were not permitted to send partially used antibiotic eye drop bottles home with patients, even when the bottle had been opened exclusively for that patient. As a result, 80%–100% of the medication was discarded at certain sites, forcing patients to repurchase the same drug at their local pharmacy—leading to unnecessary costs and inefficiencies in care delivery [55].

### 2.4. Carbon Footprint: From Patient Travel to Supply Chains and Surgical Procedures

The carbon footprint refers to the total GHG emissions generated by a product, process, or service throughout its life cycle, including production, transportation, usage, and disposal [55]. The healthcare sector is a major contributor to GHG emissions, with the U.S. healthcare system alone responsible for approximately 9% of the country's total emissions [56, 57]. Within ophthalmology, cataract surgery has a considerable carbon footprint, primarily due to patient transportation, energy-intensive surgical procedures, and emissions from medical supply chains.

Cataract surgery generates substantial carbon emissions, largely due to energy consumption, sterilization, and medical waste. A study conducted in Cardiff, United Kingdom, estimated that a single cataract surgery had a carbon footprint of 181.8 kg CO_2_eq. Extrapolating this data to national levels, researchers estimated that the 343,782 cataract surgeries performed in England in 2011 contributed approximately 63,000 metric tons of CO_2_eq [27]. Similarly, in Spain, a cataract surgery at Ávila Hospital generated 86.62 kg CO_2_eq, with 85% of emissions attributed to medical and pharmaceutical equipment [31].

A study from France reported that disposable surgical items alone accounted for 73.32% of emissions per procedure, contributing 59.49 kg CO_2_eq. Additionally, the sterilization of phacoemulsifier handpieces contributed 2.12 kg CO_2_eq per surgery [29]. In New Zealand, cataract surgery was estimated to produce 152 kg CO_2_eq per procedure—the equivalent of burning 62 L of gasoline, requiring 45 square meters of forest to offset the emissions over a year [30].

Procurement is the largest contributor to carbon emissions in cataract surgery, responsible for over 50% of total emissions. Morris and colleagues in their study found that procurement contributed 97.8 kg CO_2_eq per procedure, with medical equipment accounting for 32.6% and pharmaceuticals for 18.0% of the total emissions [27].

Another significant portion of cataract surgery's carbon footprint comes from patient travel. This is applicable to both cataract and refractive surgery. The treatment pathway typically requires multiple hospital visits, including initial referral, preoperative assessment, surgery, and postoperative follow-ups [44]. Patients and their accompanying relatives often make three to five round trips, increasing transport-related emissions. Previous studies have reported that patient travel alone accounted for nearly 9%–18% of the total carbon footprint per procedure. Given the high volume of cataract and refractive surgeries performed globally, these cumulative emissions are substantial [27].

## 3. Existing Sustainable Practices

### 3.1. Reusable Alternatives for Surgical Tools, Biodegradable Packaging, and Initiatives to Reduce Waste

Efforts to reduce single-use surgical products have gained significant traction, with institutions such as the Aravind Eye Care System (AECS) demonstrating the feasibility of reusing surgical tools and pharmaceutical supplies without compromising patient safety. By optimizing surgical efficiency and maximizing cost-effectiveness, AECS has successfully incorporated the reuse of various materials, including phacoemulsification tips, tubing, metal blades, cannulas, sutures, viscoelastic substances, intraocular drugs, and perioperative drops. Despite their widespread reuse of items typically restricted to single use in countries such as the United States, their postoperative POE rates remain comparable to or even lower than those reported in high-resource settings [36, 39]. A retrospective study analyzing over two million cataract surgeries performed across AECS's 10-hospital network over an 8-year period found that the routine use of intracameral moxifloxacin prophylaxis was associated with a 3.5-fold reduction in the POE rate, decreasing from 0.07% to 0.02% [36]. This rate is even lower than the 0.04% POE rate reported in the United States by the American Academy of Ophthalmology (AAO)'s Intelligent Research in Sight (IRIS) Registry [59]. These findings challenge the assumption that single-use-only policies are necessary to prevent infections and highlight the potential benefits of well-regulated reuse practices.

Among the surgical instruments with potential for safe reuse are phacoemulsification tips. A study assessing their durability found no ultrastructural differences in eight different types of phacotips after five uses in porcine models [37]. Each tip, regardless of whether it was originally labeled for single use or multiple use, was employed in five cataract surgeries under extreme conditions using 100% phacopower. Subsequent analysis using scanning electron microscopy (SEM) revealed no significant microscopic damage, fractures, or structural failure, suggesting that these instruments can withstand multiple uses without compromising functionality or safety [37].

Alcon's Green Innovations Surgical Team (GreenIST) has implemented measures such as replacing Styrofoam packaging with biodegradable Green Cell Foam for OVD shipments in the United States [60]. This foam, derived from U.S.-grown corn, is biodegradable, water-soluble, and multipurpose, serving as a fire starter and plant food [61]. Rayner, a global cataract surgery equipment manufacturer, has transitioned from traditional paper IFU documents to QR codes for electronic downloads, an initiative projected to save 35 tons of paper annually [62]. The company has also shifted from plastic tray and lid containers to pouches, resulting in an 83% reduction in single-use plastic packaging waste [62].

Oertli, a Swiss manufacturer of ophthalmic surgical equipment, has adopted environmentally friendly packaging solutions. Their disposable product packaging consists of PETG blisters or sterile pouches with Tyvek seals, including recycled materials, and PET-O/PP film with sterilization paper. Additionally, their products are packed in recyclable cardboard boxes, incorporating a proportion of recycled material and producing no toxic gases upon incineration [63, 64].

The Dutch Working Group on Sustainable Ophthalmology has further advocated for carbon footprint reduction in ophthalmic surgery [65]. Their recommendations include eliminating unnecessary medical devices from surgical trays, periodically reviewing tray contents to remove redundant materials, and adopting new biobased materials [65]. They also emphasize minimizing the size of surgical drapes and eye sheets, favoring reusable over disposable instruments (such as I/A handpieces, rhexis forceps, microscope caps, and steel bowls), and promoting the multiple uses of phacosleeves, filling chambers, and phaconeedles where feasible [65].

These efforts underscore the potential for sustainability in ophthalmology through strategic reuse, innovative packaging, and environmentally conscious surgical practices while maintaining patient safety and surgical efficacy.

### 3.2. Energy-Efficient Technologies and OR Sustainability

Femtosecond laser-assisted cataract surgery (FLACS) has demonstrated a significant reduction in the energy required for lens emulsification compared to conventional phacoemulsification. Studies indicate that FLACS decreases US energy consumption during quadrant removal, particularly in medium-density nuclear cataracts [66]. When assessing different phases of nuclear cataract removal, FLACS consistently exhibited lower US energy use than traditional phacoemulsification surgery (CPS), highlighting its efficiency and potential to reduce thermal damage to ocular tissues [66]. However, although FLACS offers significant intraoperative advantages, its sustainability needs further assessment, particularly regarding its environmental footprint and energy consumption associated with the laser equipment.

Further research compared three different phacoemulsification machine configurations using the Alcon Legacy 20000 and found that the NeoSoniX-incorporated machine reduced ultrasonic energy power expenditure by 27.5% across all nuclear density grades compared to standard and Advantec configurations [67]. These findings suggest that optimizing machine design can significantly enhance energy efficiency in cataract surgery.

Another study evaluating the environmental and financial impacts of phacoemulsification cassette systems found that machines using reusable cassettes offered substantial sustainability benefits over those relying on single-use cassettes [38]. The reusable cassette system resulted in a 75.3% reduction in plastic waste (306.7 kg per 1000 surgeries) and a 67.7% decrease in required storage volume (2494 m^3^ per 1000 surgeries) [38]. Additionally, the system reduced costs by €54.16 per 10 procedures and improved surgical efficiency by cutting priming time by 7 min per 10 cases, minimizing downtime between surgeries [38].

Efforts to improve OR sustainability have included the introduction of centralized sterilization departments, as advocated by the Dutch Working Group on Sustainable Ophthalmology using the EyeSustain initiative. By sterilizing instruments internally, hospitals can eliminate transport costs, reduce defective instrument rates, and ensure faster equipment turnaround. This system not only enhances operational efficiency but also significantly reduces carbon emissions associated with medical waste disposal and transportation [65].

### 3.3. Multidose Medications, Reduction in Overprescription, and Reassessment of Standard-of-Care Protocols

Sustainable pharmaceutical practices in ophthalmology aim to minimize waste using multidose eye drop bottles, reducing overprescription, and optimizing medication management. The OICS Task Force—comprising representatives from the American Society of Cataract and Refractive Surgery (ASCRS), AAO, AGS, and OOSS—has issued recommendations emphasizing the safe and efficient use of multidose topical medications [32]. These guidelines state that multidose eye drops can be safely used for multiple patients in surgical settings if proper protocols are followed, remain viable until their manufacturer-labeled expiration date when stored correctly, and should be made available for patients to take home for postoperative use whenever feasible [32].

Despite these recommendations, some healthcare professionals remain hesitant due to concerns about cross-contamination. However, studies have shown that multidose bottles, when handled properly, do not increase infection risk [39, 40]. Research from Jensen et al. demonstrated significant cost savings per cataract surgery without adverse outcomes, whereas the AECS in India reported infection rates comparable to developed countries despite using shared bottles of antibacterial eye drops, anesthetics, and balanced salt solutions throughout the operating day [41].

Regulatory authorities also support the safe reuse of multidose ophthalmic medications. Wiley Chambers, MD, director of the FDA's Division of Ophthalmology, has stated that unless labeled as single use, ophthalmic products are intended for multiple-patient use and can be safely administered until their expiration date if stored as directed [68]. A survey by the OICS Task Force found that 98% of ophthalmologists were either already using or open to using multidose bottles for perioperative patients. Commonly shared medications included mydriatics, antibiotics, nonsteroidal anti-inflammatory drugs, anesthetics, and intraocular pressure-lowering agents [69]. However, proper handling is essential—any bottle with a contaminated tip should be discarded immediately to prevent microbial transmission [69].

Efforts to improve medication sustainability have also driven legislative changes. A survey by the Illinois Society of Eye Physicians and Surgeons (ISEPS) found that only 40% of medications ordered from an on-site pharmacy and 30% from external pharmacies could be taken home by patients postoperatively [70]. In response, Illinois Senate Bill 0579 was introduced, allowing partially used topical drops from ORs and emergency departments to be dispensed for postdischarge use, with physicians assuming responsibility for medication counseling [71]. Additionally, patterns of multidose bottle reuse vary by practice setting, with surgeons at ASCs significantly more likely to adopt this approach than those in hospital outpatient departments [72].

Another strategy to optimize pharmaceutical use and minimize waste is the combination of multiple ophthalmic drugs into a single bottle. Some surgical centers have explored compounding medications to enhance efficiency and improve patient adherence. For instance, a study comparing a fixed-dose combination of gatifloxacin 0.3% and prednisolone 1%, with separately administered gatifloxacin and prednisolone, found that the combination therapy was equally effective in preventing infection and controlling inflammation after cataract surgery [73]. Similarly, another study evaluated the efficacy of a Tri-Moxi-Vanc intraocular solution (administered transzonularly) versus a topical regimen of Pred-Moxi-Ketor followed by Pred-Ketor, with results indicating comparable surgical outcomes between the two approaches [74].

However, in a recent meta-analysis of over 1 million eyes, it was found that postoperative topical antibiotics may be unnecessary when intracameral antibiotics are used, as no additional benefit in preventing endophthalmitis was observed [33]. This finding has important implications for clinical practice, antimicrobial stewardship, and patient care. By minimizing unnecessary antibiotic use, the risk of antimicrobial resistance can be reduced, supporting more sustainable practices. Additionally, standardizing the use of IC antibiotics in cataract and refractive surgery could enhance patient safety, reduce healthcare costs, and improve patient adherence to postoperative care.

A study recently found that the immediate sequential bilateral cataract surgery approach has a safety and efficacy profile that is acceptable and comparable to that of the delayed sequential bilateral cataract surgery approach [34]. This opens a topic of discussion regarding the potential advantages of immediate sequential surgery, such as reduced costs and shorter recovery times, while highlighting the need for further research to fully assess its safety and long-term outcomes.

### 3.4. Sustainability in Refractive Surgery: What Do We Know?

There is a notable lack of comprehensive studies that accurately quantify the environmental impact of traditional vision correction strategies—such as eyeglasses and contact lenses—compared to refractive surgery. Contact lenses, particularly daily disposable ones, are frequently recommended due to their apparently lower but still significant risk of infection [75, 76]. However, their environmental impact is considerable, as they contribute significantly to plastic waste through both the lenses themselves and their packaging and their discard. A recent study has identified for the first time contact lenses, particularly silicone hydrogel lenses, as a significant source of plastic and microplastic pollution [77].

Therefore, a data-driven approach is essential to understand and mitigate the environmental impact of refractive and ophthalmic surgeries. Life cycle analysis (LCA) can provide a comprehensive assessment of surgical products, whereas carbon footprint assessments quantify emissions at each stage [78]. LCA is a systematic method used to assess the environmental impact of a product, process, or service throughout its entire lifespan. This includes every stage—raw material extraction, manufacturing, transportation, usage, and disposal—allowing researchers to identify key areas for reducing waste, emissions, and resource consumption [79]. Standardized LCA methodologies should be integrated into regulatory frameworks and industry practices, ensuring that future innovations prioritize both clinical efficacy and environmental responsibility.

We believe that LCA studies are essential to rigorously assess and further promote safe and optimized vision correction strategies from a sustainability perspective. Although the environmental footprint of cataract surgery has been increasingly studied, there remains a substantial gap in the refractive procedures. The global market for refractive surgery devices was estimated at around USD 170 million in 2020 and is expected to grow at an approximate compound annual growth rate (CAGR) of 8% through 2028 [80]. Given the high global prevalence of laser-based techniques, it is crucial to evaluate their long-term ecological impact, particularly in comparison with the cumulative resource consumption and waste generation associated with glasses and contact lenses. These alternatives require continuous manufacturing, transportation, packaging, and disposal throughout a patient's lifetime, factors that may outweigh the one-time resource use of refractive surgery.

Could laser-based or intraocular refractive surgery, despite its initial resource use, represent a more sustainable vision correction strategy when compared to the cumulative environmental impact of lifelong eyeglass and contact lens production, packaging, and disposal? A more comprehensive understanding of these dynamics could provide critical insights into whether refractive surgery presents a net sustainability advantage, ultimately informing environmentally responsible clinical practices and policy decisions.

## 4. Future Perspectives

### 4.1. Eco-Friendly and Green Systems

Efforts to minimize waste and reduce environmental impact in ophthalmology have led to various sustainable initiatives across hospitals, surgical practices, and manufacturing processes. Water conservation remains a significant focus, particularly in large hospitals, where regulations in India mandate on-site wastewater treatment plants for facilities with over 100 beds. At its Pondicherry location, the AECS has implemented a decentralized wastewater treatment system that repurposes treated water for nonclinical applications such as gardening and toilet flushing while also contributing to green space development [41]. Similarly, a study by Javitt et al. assessed the economic and environmental impact of switching to alcohol-based hand scrubs for surgical preparation. Their findings indicated that this transition resulted in annual water savings of approximately 61,631 L per OR, with potential cost reductions ranging from $280,000 to $348,000 per OR per year [42].

Research suggests that the use of protective coats for patients during cataract surgery offers no proven benefits in infection prevention, making them redundant. Likewise, the requirement for shoe covers when transporting patients to the OR has been deemed unnecessary, allowing for waste reduction without compromising patient safety.

Menicon has taken significant strides toward sustainability by installing 550 solar panels at its Emmen plant in the Netherlands, generating approximately 15% of the facility's annual energy needs, equivalent to 150 MWh of electricity [81]. Additionally, Menicon has implemented eco-friendly packaging for its products, including the “Miru 1day Menicon Flat Pack,” which has reduced plastic usage by 80% compared to traditional lens containers by using 100% recycled materials from the manufacturing process. Similarly, its rigid gas permeable (RGP) lenses now come in sustainable packaging and recyclable mailing envelopes, further minimizing waste [81].

Johnson & Johnson has significantly increased its reliance on renewable energy, with 95% of its electricity in Europe, the Middle East, and Africa now sourced from renewable sources [82]. As of 2022, its Vision Care global headquarters in Florida operates on 100% renewable electricity, contributing to an annual reduction of 42,000 tons of CO_2_ emissions [82]. The company remains on track to achieve carbon neutrality by 2030 [83]. Meanwhile, Zeiss' Adelaide manufacturing site established a custom-built waste management plant in 2018, designed to recycle water used during production. This system effectively filters and compresses swarf waste, allowing for the reuse of coolant in production and saving over 500,000 L of water per year while reducing waste volume by a ratio of 20:1 [84].

### 4.2. Application of Artificial Intelligence (AI)

Advancements in AI and automation are revolutionizing cataract surgery by enhancing surgical efficiency, improving patient outcomes, and optimizing resource use. AI-driven voice-controlled interactive systems have been introduced into operating theaters, providing real-time access to critical patient data and procedural information [85]. These systems aid surgical teams by offering insights into ongoing procedures, including instrument usage, phase duration, and upcoming surgical steps, thereby improving situational awareness and workflow coordination [86]. Integrating this technology with electronic patient records (EPRs) further enhances safety measures, such as lens selection and adherence to standardized surgical protocols like the World Health Organization (WHO) checklist [86].

AI has also demonstrated its potential in recognizing surgical instruments and operative phases from raw video data, offering an additional layer of automation in phacoemulsification procedures [87, 88]. By streamlining intraoperative decision-making and improving efficiency, these systems contribute to better surgical outcomes and reduced procedural waste.

In addition to AI integration, robot-assisted cataract surgery is being explored to enhance precision and surgical dexterity. Studies have demonstrated the feasibility of robotic-assisted phacoemulsification, with systems such as the Da Vinci Xi robotic surgical system successfully performing simulated cataract surgeries [89]. Although these robotic platforms provide enhanced intraocular maneuverability and superior visualization, certain manual interventions, such as intraocular injections, still require human assistance [89]. Another study using an intraocular robotic interventional surgical system (IRISS) integrated with optical coherence tomography (OCT) successfully performed lens extraction on postmortem pig eyes, further validating the potential of robotic systems in cataract surgery [90].

Furthermore, mixed reality applications, including real-time intraoperative warnings, are being explored to improve surgical decision-making. These systems can identify and alert surgeons to potential complications before they arise, reducing surgical risks [91]. Additionally, AI-driven automation has the potential to optimize OR efficiency by improving case scheduling and resource allocation, ultimately enhancing patient output and reducing overall surgical costs [91].

### 4.3. Telemedicine and Remote Screening

The adoption of telemedicine and AI-driven screening in cataract care is transforming patient management by reducing hospital visits, improving efficiency, and lowering travel-related carbon emissions [92]. Traditionally, patients undergo multiple in-person visits, including referral, preoperative assessment, surgery, and postoperative follow-up. Reducing unnecessary travel through AI-based remote diagnosis, optometrist-led referrals, and virtual consultations can significantly cut emissions [35, 93–95]. A study by Gaskell A and colleagues conducted at two Scottish centers found that patients traveled an average of 33.1 miles per visit. By shifting from a conventional five-stop process to a streamlined one-stop cataract surgery model, the study estimated a reduction of 29.8 kg of CO_2_ emissions per patient, demonstrating the environmental benefits of minimizing hospital visits [35].

AI-based cataract screening has shown promising results in improving diagnostic accuracy and triaging patients remotely. Shimizu et al. developed an AI model trained on 38,320 cataract surgery video frames, which achieved an accuracy of 94.1% in diagnosing nuclear cataracts, with sensitivity and specificity comparable to ophthalmologists [96]. Similarly, Cheung et al. validated a computer-aided diagnosis (CAD) system using slit-lamp lens photographs, reporting a high correlation with the Wisconsin grading method (ICC: 0.81) [97]. Wu et al. implemented an AI-based referral system that integrates home self-monitoring, primary healthcare services, and specialized hospital care. Their AI model effectively detected referable cataracts, achieving an area under the curve (AUC) of over 91% even in challenging imaging conditions. This system triggers fast-track notifications for urgent cases, ensuring timely specialist intervention and reducing unnecessary clinic visits [98].

AI is also playing a critical role in postoperative care and follow-up. Automated AI tools have been successfully used to detect posterior capsular opacification (PCO) [99], a common post–cataract surgery complication, enabling timely triage for laser capsulotomy. Additionally, AI-driven virtual assistants, such as DORA [100] used for automated phone consultations, assess patient recovery remotely by collecting symptom reports, addressing concerns, and providing transcripts for clinicians to review [100]. These systems help reduce in-person follow-ups while maintaining patient safety and quality of care [100].

AI has the potential to play a crucial role in optimizing sustainable practices in ophthalmic surgery by improving workflow efficiency, reducing resource redundancy, and minimizing procedural waste. However, its implementation is not without environmental cost [101]. The computational infrastructure required to train, deploy, and maintain AI systems—particularly those relying on cloud-based platforms and continuous real-time data processing—demands significant energy consumption, often sourced from nonrenewable energy grids [102]. Thus, although AI may contribute to greener surgical practices in terms of process optimization, it is not a low-carbon solution. A critical evaluation of its full life cycle environmental footprint is necessary to ensure that its integration aligns with broader sustainability goals.

### 4.4. Regenerative Approaches

Recent research has explored pharmacological interventions aimed at delaying or reversing cataract formation, potentially reducing the need for surgical intervention. A study published in 2015 by Zhau et al. highlighted the role of lanosterol in preventing lens protein aggregation and mitigating cataract development. Lanosterol was found to decrease protein aggregation in mutant crystallin proteins in cell cultures and enhance lens clarity in animal models. Its amphipathic properties allow it to integrate into hydrophobic protein aggregates, restoring their solubility and reducing cataract severity [103].

Similarly, bendazac lysine, an aldose reductase (AR) inhibitor, has demonstrated protective effects on lens proteins. Long-term oral administration (500 mg three times daily) has been reported to help maintain lens transparency, and a double-masked placebo-controlled trial showed that 0.5% bendazac lysine eye drops, applied three times daily, could slow cataract progression [104]. Another potential therapeutic agent, 5-cholesten-3b,25-diol (25-hydroxycholesterol), functions as a pharmacological chaperone by stabilizing native crystallin proteins and activating heat shock protein 27 (HSP27), which may prevent misfolding and maintain lens protein solubility [105]. Additionally, rosmarinic acid has shown promise in delaying cataract formation and reducing lens opacity in animal models. Its efficacy in ex vivo assays suggests its potential for broader screening as a pharmacological treatment for cataracts [106].

These findings highlight the potential of nonsurgical interventions in managing cataract progression. However, although these interventions may reduce the long-term need for surgical procedures—and the associated surgical waste—they are not without environmental implications. Widespread use of such pharmacological agents, particularly if administered chronically over years or decades, would necessitate mass production, distribution, and disposal of medications and packaging. This raises concerns regarding pharmaceutical overuse, microplastic generation, and improper drug disposal, all of which contribute to environmental contamination of water systems and ecosystems. In light of these considerations, it is essential that future research endeavors not only focus on improving the clinical efficacy of treatments but also integrate environmental impact assessments early in the development process.

## 5. The Role of Policy and Scientific Community

Recognizing the environmental impact of ophthalmic procedures, organizations such as the AAO and the Royal College of Ophthalmologists are actively advocating for sustainable practices and regulatory reforms. The AAO has joined “EyeSustain,” a digital platform established in collaboration with the ASCRS and the European Society of Cataract and Refractive Surgeons, to educate the ophthalmology community on reducing environmental waste and fostering sustainable practices [107]. EyeSustain's mission is fivefold: to educate the ophthalmic community on sustainable practices, collaborate with industry to reduce surgical waste and carbon emissions, support research and innovation for minimizing ophthalmology's environmental impact, advocate for policies addressing climate change's effects on public health, and drive partnerships with industry to implement eco-friendly solutions [108].

Additionally, the AAO's “Sustainability Task Force” [109], led by Dr. Jeff Pettey at the University of Utah, is focused on promoting eco-friendly practices across ophthalmology. Another initiative, “My Green Doctor” [110], provides clinics, surgical centers, and medical offices with resources to integrate sustainability into practice management.

Similarly, the Royal College of Ophthalmologists, through its Net Zero Working Group, is spearheading efforts to decarbonize eye care [111]. This includes developing guidelines for sustainable practices, raising awareness of low-carbon healthcare solutions, and integrating sustainability into ophthalmic education. These collective efforts emphasize the need for regulatory frameworks that support environmentally responsible ophthalmic care. Some major ophthalmic manufacturers have already taken significant steps toward sustainability, as highlighted in this review.

## 6. Conclusion

Sustainability in ophthalmic surgery has become increasingly important due to the significant waste, energy consumption, and pharmaceutical disposal associated with cataract and refractive procedures. These surgeries contribute notably to emissions and medical waste, underscoring the need for eco-friendly solutions. Strategies such as minimizing single-use materials, adopting energy-efficient technologies, and using sustainable packaging are critical. Although some manufacturers have initiated green practices, a substantial gap remains between current efforts and sustainability standards that are practical and effective across diverse healthcare systems, including low-resource settings. Regulatory bodies, healthcare institutions, and the scientific community must lead policy changes and foster interdisciplinary collaboration. Clinicians play a key role by advocating for reusable instruments, multidose medications, and improved waste management. Further research is needed to establish standardized life cycle assessments and sustainability guidelines that can help redefine the standard of care, globally. Collaboration among ophthalmologists, engineers, industry, and environmental scientists is vital to advancing sustainable innovations without compromising patient care.

## Figures and Tables

**Table 1:** Studies identifying sustainability challenges and proposed solutions.

Study	Topic	Sustainability issue	Solutions	Ref
Khor et al. (Singapore, 2020)	Waste from phacoemulsification	Each surgery produced 0.814 kg (experienced surgeons) to 1.086 kg (trainees) of waste	Noted ∼60% of general waste was recyclable, indicating a substantial opportunity to reduce waste via recycling and training	[25]
Winklmair et al. (Austria, 2023)	Cataract pack material standardization	Identified 55 different cataract pack configurations (average of 0.7 kg each; drape sizes varied by 71%)	Standardizing to the lightest necessary pack could reduce cataract surgery-related CO_2_ emissions by ∼34%	[26]
Morris et al. (United Kingdom, 2013)	Carbon footprint of cataract surgery in the United Kingdom	181.8 kg CO_2_ equivalent (CO_2_e) emitted per phacocataract surgery	Procurement (production of equipment and supplies) contributed ∼97.8 kg CO_2_e per case (with ∼32.6% from medical equipment and 18.0% from pharmaceuticals), highlighting supply chain as a major target for reduction	[27]
Stern et al. (France/United Kingdom, 2024)	Paper waste due to the instructions for use	The mean and standard deviation of the weight for overall IFUs, classic IFUs, and e-IFUs were 17.6 ± 13.8, 23.5 ± 13.2, and 2.9 ± 1.9 g, respectively	The classic IFUs in IOL packaging result in a significant amount of paper waste annually, highlighting the urgent need for a rapid transition to e-IFU technology	[28]
Ferrero et al. (France, 2022)	Carbon footprint of cataract surgery in France	Each cataract procedure emitted ∼81 kg CO_2_e	Disposable surgical items accounted for ∼73.3% of emissions (∼59.5 kg CO_2_e per surgery). Reducing disposable usage (or switching to reusables with efficient sterilization) can significantly cut the carbon footprint	[29]
Latta et al. (New Zealand, 2021)	Carbon footprint of cataract surgery in New Zealand	Estimated the carbon footprint of a single cataract operation at ∼152 kg CO_2_e	Need for sustainability efforts such as energy efficiency, waste reduction, and reusable instruments	[30]
Pascual-Prieto et al. (Spain, 2023)	Carbon footprint of cataract surgery in Spain	Reported a carbon footprint of ∼86.6 kg CO_2_e for a cataract surgery	∼85% of these emissions were attributed to medical and pharmaceutical equipment, suggesting that greener manufacturing and supply chain improvements are key to reduction	[31]
Palmer et al. (OICS Task Force, United States, 2022)	Multisociety eye drop waste guidelines	Disposal of mostly full eye drop bottles after each patient due to single-use policies	An ophthalmic coalition (AAO, ASCRS, etc.) issued guidelines affirming that multidose topical eye drops can be safely used for multiple patients in surgical settings. They advised that drops can be used until expiration and should be made available for patients to take home post-op	[32]
Passaro et al. (Italy/United Kingdom, 2025)	Postoperative therapy	Routine use of postoperative antibiotic eye drops, even when intracameral antibiotics are used during surgery	Meta-analysis of over 1,000,000 eyes found no added benefit in using post-op topical antibiotics if an intracameral antibiotic was given during cataract surgery. This suggests that routine post-op antibiotic drops can be safely omitted in such cases. Implementing this change would reduce drug overuse, cut costs, and waste	[33]
Aiello et al. (Italy/United Kingdom/United States, 2023)	Immediate bilateral cataract surgery	Separate-day surgeries for two cataracts double patient visits, energy use, and resource consumption	Immediate sequential bilateral cataract surgery (ISBCS) (operating on both eyes in one session) is as safe and effective as the conventional delayed two-session approach. ISBCS can drastically reduce the number of clinic visits, travel, and perioperative resources required per patient	[34]
Gaskell et al. (United Kingdom, 2001)	Preoperative assessment	Multiple pre-op visits for cataract patients leading to repeated travel emissions	Implemented a direct optometrist referral and one-stop cataract surgery assessment clinic, resulting in an estimated 29.8 kg reduction in CO_2_ emissions per patient	[35]
Haripriya et al. (India, 2019)	Large-scale validation of antibiotic prophylaxis and reuse in cataract surgery	Reducing the need for single-use supplies	The endophthalmitis rate fell 3.5-fold—from 0.07% to 0.02%, with regular use of intracameral moxifloxacin. This sustained low infection rate, even with widespread reuse of supplies, demonstrates that effective prophylaxis enables safe, sustainable high-volume surgery	[36]
Tsaousis et al. (United States/Greece, 2018)	Multiple use of phacotips	Guidelines label phacoemulsification tips as single use, generating unnecessary waste	Postuse scanning electron microscopy showed no significant structural damage or fractures on any tips, thus concluding that phacotips can withstand multiple uses without performance loss, supporting their safe reuse to reduce instrument waste	[37]
Kallay et al. (Canada, 2024)	Reusable vs. single-use phacocassettes	Large plastic waste and cost from disposable phacocassettes used in cataract surgery	Switching to reusable cassettes led to a ∼75.3% reduction in plastic waste (∼307 kg less plastic per 1000 surgeries) and a 67.7% decrease in storage volume needed	[38]
Jensen et al. (United States, 2014)	Safety of sharing multidose drops	Fears that using the same bottle of drops on multiple patients could cause infections	With proper handling (avoiding bottle-tip contact, etc.), there was no increase in infection risk	[39, 40]
Thiel et al. (India, 2020)	Hospital wastewater recycling	Large ophthalmic hospitals generate substantial wastewater from clinical and nonclinical operations	Implemented a decentralized wastewater treatment system on-site. The treated water is reused for gardening, landscaping, and toilet flushing rather than being discarded	[41]
Javitt et al. (United States/Israel, 2020)	Waterless surgical hand scrub	Traditional presurgical hand scrubbing wastes water and time and incurs high costs	Switched from water-and-antiseptic hand scrubbing to alcohol-based hand rubs in a high-volume eye surgery center. This change saved ∼61,631 L of water per OR per year and yielded an annual cost reduction of $280k–$348k per operating room	[42]
